# Attitude of cancer patients from online self-help groups towards physical activity

**DOI:** 10.1007/s00432-020-03190-1

**Published:** 2020-03-26

**Authors:** Imke Roth, Clara Dubois, Thorsten Schmidt, Jutta Hübner

**Affiliations:** 1 Klinik für Innere Medizin, Universitätsklinikum Jena, Am Klinikum 1, 07747 Jena, Germany; 2 Krebszentrum Nord, CCC, Universitätsklinikum Schleswig-Holstein, Campus Kiel, Arnold-Heller-Straße 3, Haus 14, 24105 Kiel, Germany

**Keywords:** Physical activity, Cancer, Self-efficacy, Patient activation, Predictors

## Abstract

**Purpose:**

Physical activity (PA) is important for cancer patients during and after therapy with respect to reducing side effects and improving quality of life. The aim of the study was to examine how physically active German cancer patients are and to identify predictors for PA. In addition, patients were asked about their attitude towards PA.

**Methods:**

A questionnaire was passed on to members of self-help groups. Multiple regression analyses were run to examine possible predictors such as self-efficacy, patient activation, gender, previous PA, therapy status, and age for PA.

**Results:**

62% of the participants followed the official recommendations by the American Cancer Society for weekly aerobic activity. Multiple regression analyses could confirm age as a predictor for total PA. Higher self-efficacy and patient activation were associated with lower disease burden and a more positive attitude towards PA.

**Conclusion:**

This study contributes to the minor knowledge about PA among cancer patients. The examined group showed that there is potential for improvement regarding PA, although the majority had a positive attitude towards PA. Because of the small sample size existing of online self-help group members, results should be taken with caution.

## Introduction

### Physical activity

Physical activity (PA) plays an important role in cancer therapy for primary and relapse prevention. Furthermore, PA can have positive effects in every therapy phase. It can improve physical functioning and reduce therapy-related side effects such as fatigue (Hayes et al. 2013; McNeely et al. 2006). Furthermore, increased post-surgery PA is associated with better recovery (van Zutphen et al. 2017). In contrast, inactivity can weaken the skeleton and can lead to muscle loss as well as overweight. Being physically active has a significant association with health-related quality of life (Blanchard et al. 2008; Ferrer et al. 2011; McNeely et al. 2006). In addition, a meta-analysis by Schmid and Leitzmann showed that post-diagnosis PA is associated with a reduced risk of total mortality among breast and colorectal cancer patients, especially when patients engage in moderate activity of at least 150 min a week (Schmid and Leitzmann 2014). Similar results could also be found in patients with prostate cancer (Friedenreich et al. 2016; Kenfield et al. 2011). Another study including three different cohorts of breast cancer survivors found that low post-diagnosis PA increases all-cause mortality and breast cancer-related mortality (Nelson et al. 2016). Furthermore, PA can help to prevent the development of common comorbidities such as cardiovascular diseases (Lear et al. 2017) by decreasing insulin levels, reducing adiposity and decreasing the level of sex hormones and inflammation markers (McTiernan 2008). The risk-reducing effect does not depend on the type of PA but on the duration and intensity of the activity. A guideline for nutrition and PA from the American Cancer Society suggests 150 min of moderate or 75 min of vigorous weekly aerobic PA for cancer patients (Rock et al. 2012). An observational study among US cancer survivors found that only 12.6% of the cancer survivors were sufficiently active, respectively, meeting the guideline criteria and that older, female and obese cancer survivors were the least physically active (Loprinzi et al. 2013a). Furthermore, a few studies concerning breast cancer patients proclaim that PA levels decrease after diagnosis and do not return to the pre-diagnosis level (Devoogdt et al. 2010; Irwin et al. 2003). To our knowledge, German studies on PA in cancer patients are rare. A study by Höh et al. reported that the majority (68%) of examined cancer patients were physically active at least 3 days a week (Hoh et al. 2017). However, in the study patients were only asked how many times a week, they engaged in some kind of physically straining activity. The actual intensity of the activities was not measured.

### Patient activation and self-efficacy

A potential predictor of PA might be patient activation. Patient activation describes a patient’s engagement in health care. Patients show various ways to deal with their disease. They differ in individual skills, confidence, and knowledge in managing their own health. Some are relatively passive; others have confidence and knowledge to self-manage their health behaviors. To participate actively in one’s own medical care is essential for effective management of chronic diseases, e.g., cancer, and is essential for good compliance. Moreover, better health status and outcome are reported for patients with high patient activation (Jessica Greene et al. 2015). Cancer patients with high patient activation are more likely to eat a healthier diet, to deal with side effects efficiently and to be satisfied with their treatment by feeling better informed and understanding their diagnosis (Hibbard et al. 2017). In addition, a study by Hibbard et al. showed that positive changes in patient activation of patients with chronic disease were positively related to self-managed health behaviors such as regular exercise (Hibbard et al. 2007). Self-efficacy developed by Bandura (1994) is another concept that could possibly affect PA in cancer patients. Bauman et al. reported self-efficacy as a determinant for PA in a healthy population (Bauman et al. 2012). Self-efficacy represents the general ability to deal with difficult challenges and the level of confidence in being able to solve problems autonomously. It is a personal characteristic that might help to overcome barriers regarding PA and increase PA. A cancer diagnosis is a life-changing event and is often perceived as an extreme burden. This burden might be decreased by better self-efficacy and patient activation, since both characteristics are negatively correlated with depression (Ding et al. 2017; Goodworth et al. 2014).

### Attitude towards PA

Referring to a qualitative study examining cancer patients’ views about PA, most participants are aware that PA has benefits relating to cancer and is good for general health. Furthermore, most have the desire to increase their PA (Smith et al. 2017). Potentially, there might be a relation between the attitude towards PA and performed PA. Höh et al. reported that being rarely physically active was associated with uncertainty about the positive effects of PA such as increased well-being or better coping with the disease (Hoh et al. 2017).

### Aim

The aim of this study was to evaluate how physical active German cancer patients are and to identify relevant determinants of PA such as self-efficacy and patient activation. Furthermore, the goal was to explore cancer patients’ attitudes towards PA and their satisfaction with their performed PA. With these data, more effective interventions may be developed and target groups for sport interventions may be defined in more detail.

## Methods

### Study design, participants and material

The survey was conducted over a period of 5 months from March to the middle of August 2018. The online questionnaire was passed on via email to members of the following self-help groups with online services: “Hautkrebs-Netzwerk Deutschland e.V.” (English: Skin cancer network Germany) and “Das Lebenshaus e.V.” (English: House of life). Furthermore, the link to the online questionnaire was shared via Facebook with group members of “Unterstützung bei Krebs” (English: Cancer Support). The objective of the survey was clearly stated and the participants were informed that participation was voluntary and anonymous. For Demographics see Table 1.

**Table 1:** Demographics

	Total	in %
Gender		
Female	115	89.1
Male	14	10.9
Age (median 50 mean 48.6)		
< 40 (27–39)	21	16.3
40–49	42	32.6
50–59	57	44.2
> 60	9	7.0
Category		
Patient under treatment	57	44.2
Patient post treatment	72	55.8
Patient in employment	68	52.7
Patient unemployed	61	47.3
Cancer type		
Breast	42	32.6
Malignant melanoma	38	29.5
Sarcoma^a^	15	11.6
Gynaecological^b^	8	6.2
Gastrointestinal^c^	4	3.1
Others^d^	22	17.1

^a^Including GIST

^b^Ovarian-, cervix- cancer

^c^Rectal-, stomach-, colon- cancer

^d^Others = thyroid-, lung-, bladder-cancer, NHL, glioblastoma, basal cell-, squamous cell-, renal cell carcinoma, solitary fibrous tumor, histiocytoma

### Questionnaire

The questionnaire consisted of demographic data (gender, age, therapy status, cancer type) and five additional sections:

### Level of PA

PA was measured using the IPAQ long-form (International Physical Activity Questionnaire). The IPAQ has been developed to measure PA in adults age 15–69 and has acceptable measurement properties with a reliability of 0.80 and validity of 0.30 (Craig et al. 2003). The IPAQ was evaluated using the Guidelines for Data Processing and Analysis (Committee IR 2005). The IPAQ collects information about the duration, frequency and intensity of PA in four domains: (1) work, (2) transport, (3) domestic and gardening, and (4) leisure time during the last 7 days. A PA was only included if it was conducted a minimum duration of 10 min. Metabolic equivalent task (MET)—minutes are calculated by multiplying durations, frequencies and MET scores for each type of activity. They combine duration and physical strain in a consistent and comparable measurement unit.

### Attitude towards PA

The attitude was assessed with 12 statements on a 3-point scale such as “PA improves my body awareness.” or “Since I felt ill every physical effort is too much for me.”, to see whether the participants had a positive association towards PA (wellbeing, better coping, reducing cancer risk) or a rather negative association (harm, barriers) and if they were satisfied with their personal PA behavior.

### Self-efficacy

To measure self-efficacy the ASKU (Allgemeine Selbstwirksamkeit Kurzskala—English: general self-efficacy short scale) developed by Beierlein et al. was used (Constanze Beierlein 2012). It consists of three items on a 5-point Likert scale. The mean over all three items is calculated, ranging from 1 to 5. The internal validity of the ASKU is reported as high (Cronbach’s *α*: 0.81–0.86) (Constanze Beierlein 2012). In this survey, the internal consistency was retested resulting in a similar value of *α*: 0.84.

### Patient activation

Patient activation was quantified using the PAM-13 (Patient activation measure 13). The questionnaire developed by Hibbard et al. consists of 13 items on a 4-point Likert scale. Following the manual, a sum score was calculated and transformed to a 0–100 ranged scale. The patient activation can be categorized into four stages: (1) not believing the patient role is important (≤ 47.0), (2) lack of confidence and knowledge necessary to take action (47.1–55.1), (3) beginning to take action to maintain and improve one’s health (55.2–72.4) and (4) taking action (≥ 72.5) (Jessica Greene et al. 2015). Internal consistency was tested with a good result of Cronbach’s *α* = 0.82. This result is similar to the reported Cronbach’s *α* of 0.84 for a German sample by Brenk-Franz et al. (2013).

### Disease burden

At last, the participants were asked about how strongly they feel burdened by the disease and its consequences using a rating scale from 0 (not at all burdened) to 10 (extremely burdened).

### Statistics

IBM SPSS Statistics 25 was used for data analysis. Two multiple linear regressions were calculated to test if self-efficacy, patient activation, age, therapy status, gender and previous PA could predict variables total PA over all domains and PA during leisure time. Since data were ordinal or significantly proved to be non-normal Spearman correlations were used to analyze relations between attitudes towards PA, patient activation, self-efficacy, disease burden and PA. *p* values < 0.05 were considered significant.

## Results

### Demographics

Among the analyzed sample (*n* = 129), the median age was 50 years and 89.1% were female. The most common cancer types were breast cancer (32.6%) and malignant melanoma (29.5%). For more information on demographics, see Table 1.

### Physical activity

**Fig. 1:**
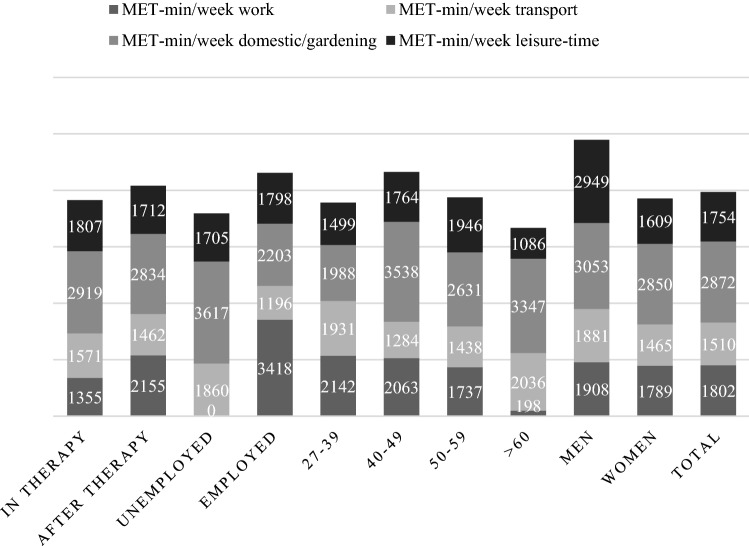
Mean MET-min/week divided by domains for therapy status, work status, age groups and gender

62% (80) of the participants followed the official recommendations by the American Cancer Society or the WHO for weekly aerobic activity which is represented by PA in leisure time (≥ 600 MET-min/week). 68.1% of participants who had finished therapy met the criteria compared to 54.4% of the patients who were currently receiving therapy. Additionally, 48.1% even met the criteria for additional health benefits in leisure time, which means they engaged in 300 min or more of an equivalent on moderate activity. The mean MET-min/week was 7937.21 MET-min/week (Mdn= 6492, SD = 5340.8), 50% had MET values between 3996 and 11828 MET-min/week. Values of MET-min/week were rather positive skewed than normally distributed (skew = 0.754; kurtosis = − 0.058). It appears that the greatest amount by 36% of the weekly activity is the result of activities in the domestic and gardening domain followed by work (23%), leisure time (22%) and transport (19%). For average MET values, see Fig. 1. The median activity time per day was 220 min (*M* = 265.30, SD = 176.01). 83.7% were physically active in their leisure time, more than half joined different activities than just walking and 89.01% used walking or bike riding as a transport possibility.

### Patient activation, self-efficacy, disease burden

The mean patient activation was 71.02 (Mdn = 72.37, SD = 17.84). More than half of the patients (58.1%, *n *= 75) could be categorized into the highest category of patient activation, meaning that they were actively taking part in their own disease management. 20.9% were beginning to take action and 10.9% were categorized into “a lack of knowledge and confidence to take action”. Only 10.1% were classified as hardly active meaning they did not believe their own role was important. The mean for self-efficacy was 3.71 (Mdn = 4, SD= 0.75). The average perceived disease burden on a scale from 0 to 10 was 6.21 (Mdn = 6, SD = 2.19). Patient activation (*r*_s_ = − 0.27, *p* < 0.01) and self-efficacy (*r*_s_= − 0.34, *p* < 0.01) were both significantly correlated with disease burden.

### Attitude towards PA

Almost 90% of the tumor patients agreed at least partially that PA improves their body awareness, their wellbeing and gave them a feeling they could do something to better cope with the disease. More than 80% were totally or partially convinced, that PA could help to reduce their risk for another tumor disease or the risk of tumor recurrence. 7.8% were worried PA could harm them. Around 20% stated they could hardly motivate themselves to be physically active. Less than 20% said they never engaged in a lot of PA and did not find a good possibility to start. 79.1% were at least partially physically active before their disease. 23.4% were completely convinced to perform enough PA, whereas 84.5% wished to increase their level of activity.

People with a positive attitude towards PA had average higher total MET values than those with a rather negative attitude, but those differences were not statistically significant. Patients with good patient activation and high self-efficacy were significantly more likely to agree with statements emphasizing a positive attitude towards PA (Statement 1, 2, 3, 4, 11) and disagreed with those statements regarding a rather negative attitude (Statement 5, 6, 7, 8, 9), see Table 2 for *p* values.

**Table 2:** Spearman Rho correlations (*r*_s_) for statements towards PA with patient activation, self-efficacy and PA (total MET-min/week)

Statement	Patient activation	Self-efficacy	PA
1. PA improves my body awareness.	0.310**	0.192*	0.126
2. PA gives me the feeling that I can do something myself to better cope with the disease.	0.415**	0.235**	0.162
3. With PA I feel better.	0.285**	0.282**	0.130
4. PA can help me to reduce the risk for another tumor disease/the risk of tumor recurrence.	0.398**	0.020	0.153
11. I think I perform enough PA.	0.301**	0.239**	0.326**
5. Through PA I feel exhausted.	− 0.229**	− 0.216*	− 0.070
6. I am afraid that PA harms me.	− 0.310**	− 0.258**	− 0.167
7. I can badly overcome to be physically active.	− 0.243**	− 0.263**	− 0.130
8. Since I felt ill every physical effort is too much for me.	− 0.365**	− 0.344**	− 0.118
9. I never engaged in a lot of PA and now I don’t find a good possibility to start with.	− 0.208*	− 0.221*	− 0.079

**p < 0.01, *p < 0.05

### Age as a predictor for PA

Two multiple regression analyses were performed to assess the influence of gender, age, self-efficacy, patient activation, previous PA, and therapy status on total PA and on leisure-time PA (MET-min/week). All predictors were analyzed for multicollinearity and autocorrelation: The variance inflation factor (VIF) ranged between 1.05 and 1.25. Durbin–Watson was close to two (2.12) indicating that multicollinearity and autocorrelation were no issues. The first model for total PA was significant with *F* (6, 122), *p* = 0.037 and *R*^2^ = 0.103 meaning the determinants included in the model accounted for approximately 10% of the variance of PA. This can be rated as a low attribute towards explained variance. Only age was a significant predictor with  = − 0.19, *p *= 0.033. The second model assessing the same predictors for PA in leisure time did not become significant (*F* (6, 122), *p* = 0.101 and *R*^2^ = 0.082). See Table 3.

**Table 3:** Multiple regression model 1: total PA and model 2: leisure time PA

	Total PA				Leisure-time PA			
	*B*	SE *B*	*β* (Beta)	*p*	*B*	SE *B*	*β* (Beta)	*p*
Constant	7938.93	4290.47		0.067	1636.21	1672.37		0.330
Self-efficacy	648.80	677.16	0.09	0.340	317.78	263.95	0.12	0.231
Patient activation	47.78	28.71	0.16	0.099	6.68	11.19	0.06	0.552
Gender	− 2468.47	1533.14	− 0.14	0.110	− 1298.73	597.60	− 0.20	0.032
Therapy status	1119.54	939.77	0.10	0.236	82.28	366.31	0.02	0.823
Previous PA	698.36	608.08	0.10	0.253	269.04	237.02	0.10	0.259
Age	− 118.98	55.11	− 0.19*	0.033*	− 21.04	21.48	− 0.09	0.329

Model 1: *R* = 0.321 *R*^2^ = 0.103 *F*(6, 122) = 2.329 *p* < 0.05, model 2: *R* = 0.287 *R*^2^ = 0.082 *F*(6, 122) = 1.82 *p* = 0.101

## Discussion

### Most participants met the recommendation for PA

The participating patients showed a very high PA in comparison to many other surveys. In the present study, 62% met the recommendation for aerobic PA which is represented by PA in leisure time. In contrast, a study on adherence to lifestyle behavior recommendations by Blanchard et al. found that only 29.6–47.3% met the PA recommendations. Skin cancer patients were closest to meeting the recommendations (47.3%) (Blanchard et al. 2008). The high levels of PA in this study might be affected by 29.5% of participants with skin cancer. Another study that measured PA by accelerometer reported that only 12.6% of an American cancer population were sufficiently active (Loprinzi et al. 2013b). Yet another study found that 95.5% of patients with a cancer history did not meet the activity guidelines (Smith et al. 2011). To our knowledge, this study is the first providing detailed information on PA in German cancer populations. In comparison to the general German population patients in this survey showed higher PA. The health report by the DKV 2018 stated that only 43% of German citizens reached the benchmark for recommended PA (Froböse and Wallmann-Sperlich 2018). However, studies using the IPAQ to measure PA reported similar results as in this study (Gerovasili et al. 2015; Lear et al. 2017).

However, it seems that we examined a relatively homogeneous highly active population. Most participants were very active, including some extreme cases and only very few patients with low levels. A reason for bias might be that the IPAQ is relatively long and asks for PA in different domains retrospectively which can lead to confusion and overreporting. Furthermore, the patients showed an overall positive attitude towards PA. Kwan et al. observed that breast cancer patients who were physically active before diagnosis remained active post-diagnosis (Kwan et al. 2012). In addition, a majority (79.1%) of our collective was already active before their disease. With respect to skin cancer, outdoor, PA is a risk factor for this type of cancer; therefore, people who practice outdoor activities are more likely suffering from skin cancer. Yet, the survey period took place through spring and summer as day length and weather conditions are known to influence PA which might lead to high reported PA (Wu et al. 2017). Regarding the tumor types in this survey skin and breast cancer, patients were highly represented, who often are younger. Ninety-three % of the participants were under 60 years. This most probably is also due to the online setting of the survey. In addition, they are treated with minor surgeries that might have a lower impact on strength and fitness and let to faster recovery. For further reasons, see “Limitations”.

### Cancer patients showed high patient activation and self-efficacy

Our collective showed comparable means for self-efficacy and patient activation to reference values [self-efficacy 3.7, reference value 4 (Constanze Beierlein et al. 2012); patient activation 71, reference value 67.5 (Zill et al. 2013)]. Although our patients showed high patient activation and self-efficacy, we could not prove them as predictors for PA. However, we could confirm our assumption that patient activation with small effect size and self-efficacy with middle effect size show negative correlations with disease burden. One explanation is that activated patients might have better coping strategies, because they know better about their disease and its consequences. Physicians should encourage these characteristics, keeping in mind that cancer diagnosis is an enormous burden for patients.

### Attitude towards PA

Most cancer patients confirmed PA as an opportunity to cope with the disease and to reduce their cancer or recurrence risk and showed readiness for PA, which is similar to other examined cancer populations (Hoh et al. 2017; Smith et al. 2017). Moreover, only around one quarter was completely satisfied with their performed PA and most of the participants (46.5%) wished to be more physically active. Yet, 25% were at least partly worried that PA could harm them. Patients proclaimed that only “little information was given from oncology health professionals on how to achieve adequate levels of PA” (Smith et al. 2017) and only one in two patients reported to feel well informed about PA (Hoh et al. 2017). Patient education towards the positive effects of PA should be improved. Cancer patients should be aware that PA is feasible and harmless and provides significant health benefits. Regular offers, e.g., rehabilitation/cancer sports groups could help patients who can badly overcome to be physically active.

### Age is a predictor for PA

Younger age increases the likeliness to engage in PA, which is in line with several studies (Bauman et al. 2012; Gerovasili et al. 2015). Although in the literature, there is evidence for relations between self-efficacy (Bauman et al. 2012; Coups et al. 2009), patient activation (McCabe et al. 2018), gender (Gerovasili et al. 2015; Loprinzi et al. 2013b; Smith et al. 2011), previous PA (Bauman et al. 2012) or therapy status (Hoh et al. 2017), and PA we could not confirm any of those as predictors for general PA nor for PA in leisure time. Moreover, the *R*^2^ of the total PA regression model was only 0.103, which means 90% of the variance of PA is explained by other factors than those included in our model. Predictors with more influence on PA might be education level, income, profession, obesity, general health status, genetics, family/friend support, living space (rural or urban) or environmental conditions such as access to sports facilities, further research should be done to show how PA can be predicted and influenced.

### Limitations

The questionnaire was only addressed to members of online self-help groups. This might explain our extraordinary results in view of PA level and attitude towards PA. Considering that patients within online self-help groups might differ in their characteristics and show a different health awareness, e.g., are more likely to engage in self-management practices such as PA, are younger and might tend to have in general higher self-efficacy and patient activation scores. Moreover, it should be considered that patients with a greater affinity to PA were more motivated to answer a questionnaire towards PA or that patients answer more positively because of social desirability. Furthermore, it was a self-reported assessment, answers can be subjective and unreliable, in general, there is evidence that self-reported questionnaires tend to over-report PA in comparison to objective measures with accelerometer (Gaede-Illig et al. 2014). In addition, those with limited access or internet aversion are underrepresented in this study. In general, the relatively small sample size, the high level of PA and the not normally distributed MET-minutes/week in our study population might limit the generalizability of our results. We did not ask exactly what therapy the patients were undergoing; therefore, we cannot assess the impact of therapy on physical fitness. Furthermore, gender was unequally distributed and previous PA was only measured by one subjective dichotomous question. As well it was a short observation period and we did not ask for education level nor for socioeconomic status. We did not ask for participants’ professions which limits the transparency of PA in the work domain.

## Conclusion

In summary, it is important that all cancer patients, especially older ones, are encouraged to maintain their PA status and to help inactive survivors to increase their PA to benefit from the positive effects of PA. To avoid potential overestimation and make it easier to compare results objective measurement methods such as an accelerometer could be used. To examine a greater and more balanced population in future studies one possibility would be to offer online surveys on tablets in hospital or medical practice waiting areas. Another idea could be to generate a general mailing list for online surveys. Regarding the study design, questions should be easy to understand and time to completion should be short to increase the number of participants and their compliance. A concept to combine long-term research in PA and motivating cancer patients for PA would be to develop a smartphone app with an accelerometer to measure PA, reminder and instructions for practical exercises (photos, videos) to motivate patients and short questionnaires towards PA. Furthermore, more research should be conducted detecting relevant predictors for PA to detect patients at risk for low PA.

## Data Availability

The data sets generated during and/or analysed during the current study are available from the corresponding author on reasonable request.
